# Drug–target interaction prediction via multiple classification strategies

**DOI:** 10.1186/s12859-021-04366-3

**Published:** 2022-01-20

**Authors:** Qing Ye, Xiaolong Zhang, Xiaoli Lin

**Affiliations:** Hubei Key Laboratory of Intelligent Information Processing and Real-Time Industrial System, School of Computer Science and Technology, Wuhan University of Science and Technology, Wuhan, China

**Keywords:** Drug–target interaction, Multiple classification strategies, Within-class imbalance

## Abstract

**Background:**

Computational prediction of the interaction between drugs and protein targets is very important for the new drug discovery, as the experimental determination of drug-target interaction (DTI) is expensive and time-consuming. However, different protein targets are with very different numbers of interactions. Specifically, most interactions focus on only a few targets. As a result, targets with larger numbers of interactions could own enough positive samples for predicting their interactions but the positive samples for targets with smaller numbers of interactions could be not enough. Only using a classification strategy may not be able to deal with the above two cases at the same time. To overcome the above problem, in this paper, a drug-target interaction prediction method based on multiple classification strategies (MCSDTI) is proposed. In MCSDTI, targets are firstly divided into two parts according to the number of interactions of the targets, where one part contains targets with smaller numbers of interactions (TWSNI) and another part contains targets with larger numbers of interactions (TWLNI). And then different classification strategies are respectively designed for TWSNI and TWLNI to predict the interaction. Furthermore, TWSNI and TWLNI are evaluated independently, which can overcome the problem that result could be mainly determined by targets with large numbers of interactions when all targets are evaluated together.

**Results:**

We propose a new drug-target interaction (MCSDTI) prediction method, which uses multiple classification strategies. MCSDTI is tested on five DTI datasets, such as nuclear receptors (NR), ion channels (IC), G protein coupled receptors (GPCR), enzymes (E), and drug bank (DB). Experiments show that the AUCs of our method are respectively 3.31%, 1.27%, 2.02%, 2.02% and 1.04% higher than that of the second best methods on NR, IC, GPCR and E for TWLNI; And AUCs of our method are respectively 1.00%, 3.20% and 2.70% higher than the second best methods on NR, IC, and E for TWSNI.

**Conclusion:**

MCSDTI is a competitive method compared to the previous methods for all target parts on most datasets, which administrates that different classification strategies for different target parts is an effective way to improve the effectiveness of DTI prediction.

## Background

Drug development is a time-consuming and expensive process that is plagued with the problem known as the high attrition rate. This led to the practitioners’ great interest in drug repositioning due to its potential to reduce the time, cost, risk and effort inherent in developing new drugs. Some drug-target interaction (DTI) prediction methods have been proposed in the past several years, which can be divided into two categorists: similarity based methods and feature based methods.

Similarity based methods mainly use the similarity relationships between samples. Some similarity based methods proposed new optimization objective functions for similarity decomposition [1–5]. Ban et al. proposed a neighborhood regularized logistic matrix factorization [1], which can utilize the neighborhood information. Cui et al. proposed a L2,1 graph regularized matrix factorization to learn flow patterns in combination with the previous matrix-decomposition method [2]. Li et al. proposed a multi-view low rank embedding to integrate multi-view representations of drugs and proteins [3]. Mongia et al. proposed a multi-graph regularized nuclear norm minimization based method for DTI, which predicts the interactions between drugs and target proteins from three inputs [4]. Wang et al. proposed an effective computational model of dual Laplacian graph regularized matrix completion, where the drug and the target similarities can be fully exploited by using a dual Laplacian graph regularization term [5].

Although designing different optimization objective functions can make the decomposition factor meet different conditions, the decomposition factor heavily depended on similarity. Some similarity based methods designed a new method to calculate the similarity [6–8]. Zong et al. calculate the similarities within linked tripartite network, which enhanced existing association discovery methods by using a topology-based similarity measure [6]. Ding et al. developed a fuzzy bipartite local model, where multiple kernels are constructed in drug and target spaces [7]. Fan et al. introduced the similarity information of drugs/targets, and proposed the neighborhood constraint to regularize the unknown cases [8]. However, because the distributions of drugs and targets are very complex, it is hardly to design a good similarity calculation method. To overcome this problem and in order to make better use of the information contained in the feature, some feature-based methods have also been proposed.

Firstly, the feature is very important for the feature based methods, and some researcher proposed new feature extraction methods to extract more features from targets and drugs [10–15]. Li et al. used rotation forest in DTI, where local phase quantization descriptors are used to extract evolutionary information in the position-specific scoring matrix (PSSM) [10]. Farshid et al. used Adaboost in DTI, where many feature extraction methods were used in the same time [11]. Jiang et al. proposed an ensemble system integrating *k* nearest neighbor classifier with a novel feature encoding scheme to identify DTI [12]. Mahmud et.al predicted DTI based on drug chemical structure and protein sequence by using extreme gradient boosting (XGBoost) with synthetic minority oversampling technique (SMOTE) [13]. Han et al. predicted DTI by using Lasso with random forest based on evolutionary information and chemical structure [14]. Xu et al. infer the DTI by using graph isomorphic network and word vector matrix [15].

Secondly, because it is unclear which feature is the best, many features could be extracted for the target and the drug in the same time [16–18], and then some dimensional reduction methods have been proposed for DTI [19–23]. Ezzat et al. proposed a framework for DTI prediction by leveraging both feature dimensionality reduction and ensemble learning [19]. Aman et al. proposed a bagging based ensemble framework named for DTI prediction by using dimensionality reduction and active learning to deal with class-imbalanced data [20]. Mahmud et al. predicted DTI based on protein features with under sampling and feature selection techniques with boosting [21]. Feng et al. proposed a supervised discriminative sparse principal component analysis [22] and a graph Laplacian sparse principal component analysis for dimensional reduction [23]

Thirdly, some new classifiers are also proposed for DTI [24–30]. He et al. presented a method called SimBoost that predicts continuous values of binding affinities of compounds and proteins and thus incorporates the whole interaction spectrum from true negative to true positive interactions [24]. Rayhan et al. proposed an ensemble model which uses extra tree as weak learners inside a boosting scheme while holding on to the best model per iteration [25]. Pliakos et al. proposed a new learning method which addresses DTI prediction as a multi-output prediction task by learning ensembles of multi-output bi-clustering trees on reconstructed networks [26]. Zhang et al. used several random projections to build an ensemble random projection tree system [27]. Buza et al. selected a random subset of features and used only the selected features when training the local models [28]. Ezzat et al. proposed another ensemble learning method that incorporates techniques to address the issues of between class imbalance and within-class imbalance [29]. Ye et al. proposed a multiple output deep neural network to enhance the deep neural network learning ability with a kind of auxiliary classifier layers [30].

Although the above methods can solve some problems from different sides, they do not solve the problem that different targets are with very different numbers of interactions. For targets with larger numbers of interactions (TWLNI), many positive samples can be generated. But for targets with smaller numbers of interactions (TWSNI), so few interactions can only produce a small number of positive samples. As a result, different classification strategies should be designed for these two types of targets. Based on the above idea, in this paper, a new DTI prediction method based on multiple classification strategies (MCSDTI) is proposed.

In MCSDTI, targets are firstly divided into TWLNI and TWSNI. For TWLNI, because drug-target interactions are very sparsely distributed in the drug-target pair space, predicting interactions for these targets together with their neighbors could introduce more negative samples than positive samples. Furthermore, these targets could own enough positive samples for predicting their interactions. So interactions of TWLNI are predicted by using their owned positive samples. For TWSNI, numbers of positive samples of targets are too small. So the positive samples of their neighbors are used together to predict their interactions. As a result, using different classification strategies in different situations can make better use of the advantages of these classification strategies. What's more, TWLNI and TWSNI are evaluated independently, as the result could be mainly determined by TWLNI when TWLNI and TWSNI are evaluated together.

The contribution of this paper can be concluded as follows:

1. As far as we known, this is the first time that interactions of TWLNI and TWSNI are predicted by different classification strategies, which can make better use of the advantages of these classification strategies in different situations.
2. TWLNI and TWSNI are evaluated independently, which can overcome the problem that the improvement for TWSNI could be overwhelmed when TWLNI and TWSNI are evaluated together.
3. Designe a new classifier and a new evaluator for TWLNI, which can overcome the negative impact of samples of the neighbors.
4. Find a good classifier for TWSNI, whose effect for TWSNI has been overwhelmed by TWLNI.
5. Provide a new research idea for DTI prediction, as interactions of TWLNI and TWSNI cannot be predicted in the same time.

The remaining of this paper is organized as follows. Section 2 introduces the Methods. Section 3 introduces the results. Finally, Section 4 gives concluding remarks.

## Methods

### Data and motivation

Five datasets are used in this work, such as nuclear receptors (NR) [31], ion channels(IC) [31], G protein coupled receptors (GPCR) [31] and enzymes (E) [31], and drug bank (DB) [32]. The simplified molecular input line entry system (SIMILES) of drugs and sequences of targets are offered by these datasets, which can be used to extract the features for drugs and targets. The simple statistics for five datasets are given in the Table 1, where the 2nd to 4th rows respectively represented the number of drugs, targets and interactions, the 5th row represented the proportion of interactions among drug-target pair space.

**Table 1:** Simple statistics for datasets

Data sets	NR	IC	GPCR	E	DB
Drugs	54	210	223	445	5877
Targets	26	204	95	664	3348
Interactions	90	1476	635	2926	12,674
Proportion	6.4%	3.4%	3.0%	0.99%	0.064%

By analyzing these datasets, two conclusions can be obtained. Firstly, drug-target interactions are very sparsely distributed in the drug-target pair space, which can be shown by the Table 1. It can be seen from the 5-th row of the Table 1 that the percentage of interactions in the drug-target pairs space are only 6.4%, 3.0%, 3.4% 0.99%, 0.064% respectively on NR, GPCR, IC, E and DB, which shows that the number of interactions is much smaller than the number of drug-target pairs.

**Fig. 1:**
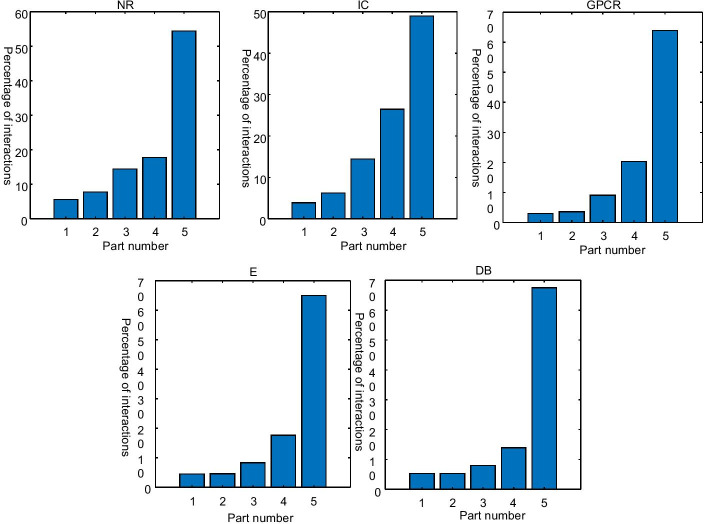
The distribution of interactions on five datasets, where Feature vector extraction

Secondly, most of the interactions focus on only a few targets, which can be shown by the Fig. 1. Distributions of interactions on four datasets are given. Targets are divided into five parts according to the numbers of interactions of targets and each part owns the same number of targets, where targets in the 1-th part owns smaller numbers of interactions, targets in the 2nd part owns larger numbers of interactions, targets in the 3rd part owns more large numbers of interactions and so on. It can be seen from Fig. 1 that more than 60% of interactions focus on 20% of targets on GPCR, E, DB, and nearly 50% of interactions focus on 20% of targets on NR and IC. And then some targets are with larger numbers of interactions, but other targets are with smaller numbers of interactions.

As a result, it is difficult to design a prediction strategy that can handle all these cases. So in this paper, different classification strategies are designed for these two types of targets.

To predict the DTI for a drug target pair, the feature vectors for the drug and the target should be firstly extracted. Some types of features have been proposed for the drugs, such as molecular substructure fingerprints, constitutional, topological, quantum chemical properties, and geometrical. Here the PubChem molecular substructure fingerprint is extracted for the drug by PaDEL [33], where the input of PaDEL is the SMILES of the drug. The extracted drug feature is defined as *D*. In this type of representation, each molecular structure is described by a Boolean vector, which is a fingerprint of a structural key according to a substructure pattern of the predefined PubChem database [34]. This feature gives a direct relationship between the molecular and properties and retain the entire structure of the drug molecule [34].

More types of features have been also proposed for targets, such as amino acid composition, dipeptide composition, autocorrelation descriptors, composition, transition, distribution, quasi-sequence-order descriptors, pseudo-amino acid composition, amphiphilic pseudo-amino acid composition, topological descriptors for atom model, total amino acid properties. In this paper, all above features are extracted for targets by Protein features (PROFEAT) [35], where the input of PROFEAT is the sequence of the target. These features can describe the target from different aspects and the dimension of these features is not very big. The extracted target feature is defined as *T*.

The simple information of the extracted features is represented in the Table 2. It can be seen from Table 2 that dimensions of the drug feature, target feature and total feature are respectively 1024, 1437 and 2461. Furthermore, it also can be seen from the 4-th row of Table 1 that number of interactions of NR, GPCR, IC, E and DB are respectively 90, 635, 1476, 2926 and 12,674. Obviously, this is a high-dimensional small sample problem, which will be considered in designing classification strategies.

**Table 2:** Simple information of the extracted features

Data sets	NR	IC	GPCR	E	DB
Drug feature dimension	1024	1024	1024	1024	1024
Target feature dimension	1437	1437	1437	1437	1437
Total feature dimension	2461	2461	2461	2461	2461

### Overview of MCSDTI

**Fig. 2:**
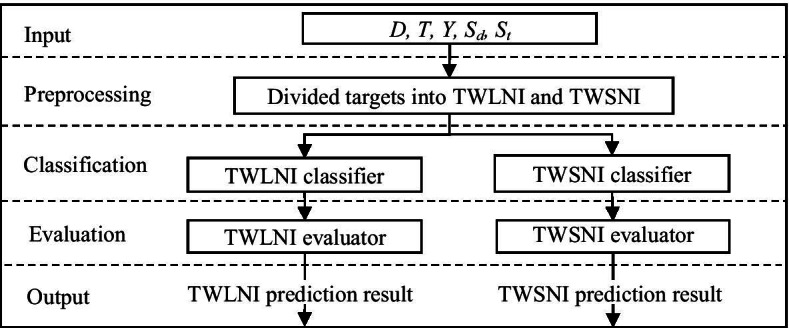
The flowchart of MCSDTI

Given drug features *D*, target features *T*, interaction matrix *Y*, drug similar *S*_*d*_ and target similar *S*_*t*_, the flowchart of MCSDTI is shown. It can be seen from Fig. 2 that MCSDTI has 5 steps, where 1st and 5th steps are the input step and the output step. Step 2 to step 4 will be simply introduced in the following.

In the preprocessing step, the targets are divided into TWLNI and TWSNI according to the number of interactions, where TWLNI contains targets with larger numbers of interactions, and TWSNI contains targets with smaller numbers of interactions. In the classification step, the TWLNI classifier and the TWSNI classifier are respectively designed for TWLNI and TWSNI, which can make better use of the advantages of these classifiers in different situations. In the evaluation step, the TWLNI evaluator and the TWSNI evaluator are respectively designed for TWLNI and TWSNI. Two evaluators are designed here, as percentages of interactions of targets with top number of interaction among all interactions are very big. And then the result could be mainly determined by TWLNI when all targets are evaluated together, which could make that the improvement for TWSNI is overwhelmed.

### TWLNI classifier and evaluator

**Fig. 3:**
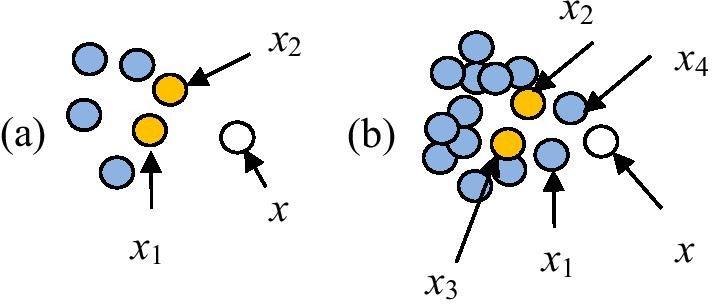
An example used to show the negative impact of samples of the neighbors

A larger number of positive samples can be generated for the TWLNI, and then there would be enough positive samples to predict the interactions of these targets. In this case, because drug-target interactions are very sparsely distributed in the drug-target pair’s space, after adding samples of neighbors, much more negative samples than positive samples would be added. And then the effect of predicting DTI for this target may be worsen, which can be shown by the Fig. 3, where Fig. 3a shows the samples of a target and Fig. 3b shows the samples after adding the samples of its neighbors, *x* is a testing sample of the target, *x*_1_ and *x*_*2*_ are two positive samples of this target, *x*_*3*_ and *x*_*4*_ are two negative samples of its neighbors. It can be seen from the Fig. 3b that many negative samples could be added around the positive samples of this target. As a result, the test sample *x* could be rightly predicted in the Fig. 3a but be wrongly predicted in the Fig. 3b.

To overcome the above problem, interactions of TWLNI are predicted by using their own positive samples in this paper. Given a training drug feature  set $D = \{ d1,d2, \ldots ,du\} \in R^{u \times p}$, training target feature set  $T = \{ t1,t2, \ldots ,tv\} \in R^{v \times q}$, and the corresponding interaction matrix $Y \in R^{u \times v}$, where *u* is the number of drugs, *p* is the number of the drug features, *v* is the number of targets, and *q* is the number of target features. To predict the interaction of *t*_*j*_, *D* can be seen as *u* samples, $Y,j$ can be seen as the corresponding class label. As a result, the pseudo code of TWLNI classifier can be shown by the Algorithm 1.
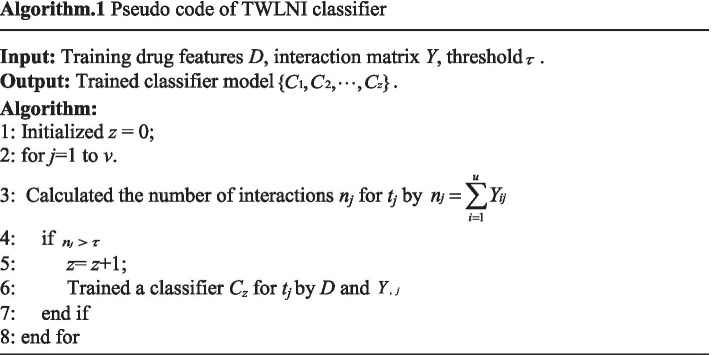


In the step 6 of Algorithm 1, classifier models can be utilized here. However, the number of positive samples is small and the dimension of the extracted feature is high, which should be considered by the utilized classifier model. By analyzing the principles of some classification models, the decision tree has the ability to deal with such problem. The decision tree is generated by a recursive method [36]. In each recursive step, a feature that can gain the most information is used to generate the child node of the decision tree. As a result, the decision tree is influenced by the number of useful features but not the total number of features

It can be seen from Algorithm 1 that this algorithm separately trains a classifier for each target. As a result, the evaluation criteria for each target should be also calculated separately. To more easily describe the evaluator, the pseudo code of TWLNI evaluator is shown in the Algorithm 2. It can be seen from the Algorithm 2 that the evaluation criteria result of *t*_*j*_ is calculated by the step 4–10, and the mean of evaluation criteria results of all targets is calculated by the step 12.
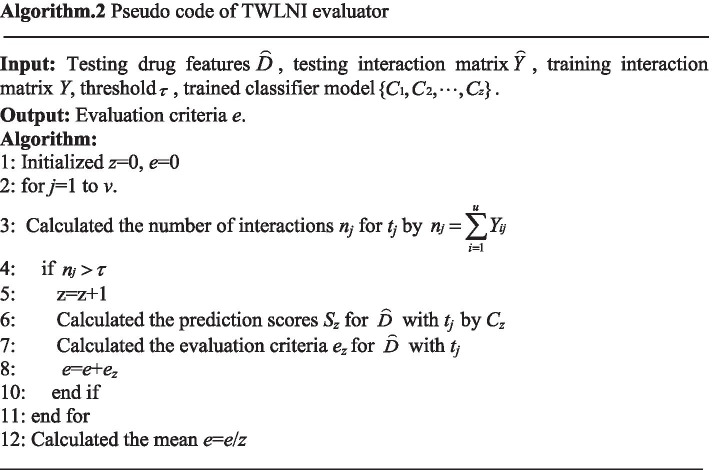


### TWSNI classifier and evaluator

Too few positive samples can be generated for TWSNI. In this case, there are not enough positive samples for this target to predict DTI, so other positive samples should be utilized to improve the effect of DTI prediction. An optional method is to use the positive samples generated by its neighbors.

However, according to the principle of clustering, neighbors of TWSNI would also with smaller number of interactions. As a result, a feature based classifier could be hardly trained in this case. To overcome this problem, a similar based method is used to predict the interactions for these targets. However, because the distributions of drugs and targets are very complex, the similarity calculated by the existing similarity calculation methods could be not good. Specially, the further away the drug or target is, the worse the similarity is. As a result, the nearest profile (NP) [31] is used to improve the DTI effect for TWSNI in this paper.

Given drug similar $Sd \in R^{nd \times nd}$, target similar $St \in R^{nt \times nt}$, and interaction matrix $Y \in R^{nd \times nt}$, where *n*_*d*_ and *n*_*t*_ are the number of drugs and targets, the interaction $Ytnew$ of a new target $tnew$ can be predicted as following [31]:

1 $$Y(:,tnew) = St(tnew,tnearest)Y(:,tnearest)$$

where $tnearest$ is the nearest target of $tnew$ and $Y(:,tnearest)$ is the interaction of $tnearest$.

The interaction $Ydnew$ of a new drug $dnew$ can be predicted as following [31]:

2 $$Y(dnew,:) = Sd(dnew,dnearest)Y(dnearest,:)$$

where $dnearest$ is the nearest target of $dnew$ and $Y(dnearest,:)$ is the interaction of $dnearest$.

Finally, the interaction $Y(dnew,tnew)$ of a drug-target pair $(dnew,tnew)$ can be predicted by mean of their scores.

The method NP is only used to evaluate the DTI effect for TWSNI. To utilize the information offered by their neighbors, all targets are used to calculate $Y(dnew,tnew)$, as there are not enough positive samples for TWSNI.

After calculating all $Y(dnew,tnew)$, only the evaluation criteria of TWSNI is output, as the result could be mainly determined by TWLNI, which could overwhelm the improvement for TWSNI. To more easily describe the processing, the pseudo code of TWSNI classifier and evaluator is shown in the Algorithm 3. It can be seen from the Algorithm 3 that the processing is not divided into training processing and testing processing, as the training processing and testing processing of the similar based method are processed in the same time.
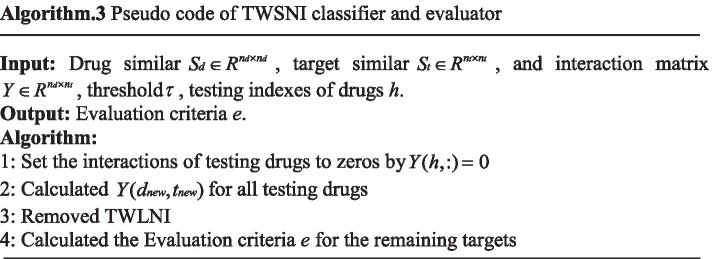


## Results

To verify the effectiveness of our proposed multiple classification strategies, our method are compared with the following methods, such as decision tree (DT)[36], random forest (RF) [36], nearest profile (NP) [31], weighted profile (WP) [31], network-based inference (NBI) [37], regularized least squares-avg (RLS) [38], regularized least squares-kron (RK) [9], ensemble decision tree (EDT) [19], ensemble kernel ridge regression ensemble (EKRR) [19] and so on.

### Experimental setting

A standard fivefold cross validation is performed and the AUC for each method (i.e. the area under the receiver operating characteristic curve) is computed. More precisely, the drugs are divided into 5 parts, where one part is used for testing and other parts are used for training. For each of the methods being compared, 5 AUC scores were computed (one for each fold) and then averaged to give the final overall AUC score. The AUC score can be biased when the data is imbalanced. However, in this paper, TWLNI and TWSNI are evaluated independently, which means that only targets with similar imbalance are evaluated together. And then imbalance does not affect the effectiveness of AUC for each method. Furthermore, AUC is a good performance evaluation metric for binary classification problem. As a result, AUC is used as the evaluation metrics in this paper.

Many parameters should be set for the compared methods. Parameters of DT, RF, EDT and EKRR used in this paper are the same as that used in the Ref. [19]. Default parameter values were used for DT and MCSDTI as defined in MATLAB’s fitctree. The number of trees should be set for RF, which is set to 50. The dimensionality reduction parameter and the number of subspaces should be set for EDT, which are set to 0.8 and 50. The dimensionality reduction parameter, the number of subspaces, the decay term, the Tikhonov regularization parameter, and an adjustable parameter should be set for EKRR, which are set to 0.2, 20, 0.7, 1 and 0.5. The decay term, the Tikhonov regularization parameter, and an adjustable parameter should be set for RLS and RK, which are set to 0.7, 1 and 0.5. NP, WP and NBI do not need to set parameters.

All methods need to extract the drugs features *D* and targets features *T*, which can be extracted by the methods described in the subsection “Feature vector extraction”. For our method, the experiments results can be obtained by Algorithm 1, Algorithm 2 and Algorithm 3. For the other compared methods, the $Y(dnew,tnew)$ for all testing drugs is firstly calculated by these methods. And then the experiment results for TWLNI are calculated by removing TWSNI and the experiment results for TWSNI are calculated by removing TWLNI.

### The experiments for TWLNI

The experiment results are presented in Table 3. These experiments would be used to answer the following questions:

1. Which threshold $\tau$ should be set for our method?
2. Is our method better than the compared methods?

**Table 3:** AUCs for TWLNI, where $\tau$ used in Algorithm 1 are respectively set to 1, 3 and 5

Dataset	$\tau$	1	3	5	Mean
NR	DT	51.90 ± 0.19	47.30 ± 0.23	47.30 ± 0.23	48.83
	RF	76.10 ± 0.40	77.30 ± 0.30	67.80 ± 0.36	73.73
	NP	69.50 ± 2.00	74.90 ± 0.37	73.90 ± 0.70	72.77
	WP	72.80 ± 0.22	80.60 ± 0.80	75.40 ± 0.10	76.27
	NBI	72.40 ± 0.30	79.70 ± 0.80	73.80 ± 0.20	75.30
	RLS	77.00 ± 0.32	87.10 ± 0.40	88.20 ± 0.49	84.10
	RK	78.60 ± 0.30	*87.80* ± *0.37*	88.50 ± 0.40	84.97
	EDT	*81.70* ± *0.42*	86.10 ± 0.10	81.20 ± 0.35	83.00
	EKRR	76.40 ± 0.35	87.40 ± 0.29	*88.70* ± *0.39*	84.17
	ours	**84.17** ± **0.48**	**90.12** ± **0.46**	**90.54** ± **0.56**	**88.28**
IC	DT	51.30 ± 0.18	53.30 ± 0.33	54.30 ± 0.34	52.97
	RF	67.70 ± 0.27	72.10 ± 0.12	72.90 ± 0.11	70.90
	NP	62.60 ± 0.10	64.30 ± 0.40	66.10 ± 0.60	64.33
	WP	*72.50* ± *0.54*	*76.60* ± *0.50*	*77.40* ± *0.13*	75.50
	NBI	72.30 ± 0.54	76.40 ± 0.40	77.20 ± 0.13	75.30
	RLS	68.30 ± 0.67	70.60 ± 0.45	71.40 ± 0.42	70.10
	RK	68.40 ± 0.57	70.50 ± 0.36	71.00 ± 0.31	69.97
	EDT	71.50 ± 0.24	76.00 ± 0.18	77.20 ± 0.35	74.90
	EKRR	69.50 ± 0.64	71.60 ± 0.38	72.50 ± 0.33	71.20
	ours	**72.50** ± **0.43**	**78.08** ± **0.54**	**79.71** ± **0.54**	**76.77**
GPCR	DT	59.90 ± 0.20	62.60 ± 0.80	62.70 ± 0.13	61.73
	RF	78.00 ± 0.18	77.60 ± 1.90	77.80 ± 0.19	77.80
	NP	67.80 ± 0.10	69.30 ± 0.40	70.30 ± 0.20	69.13
	WP	81.90 ± 0.11	80.40 ± 0.22	80.70 ± 0.20	81.00
	NBI	81.80 ± 0.11	80.40 ± 0.22	80.60 ± 0.19	80.93
	RLS	81.50 ± 0.11	81.20 ± 0.14	81.00 ± 0.15	81.23
	RK	82.00 ± 0.40	81.00 ± 0.11	80.90 ± 0.13	82.30
	EDT	*82.70* ± *0.12*	82.20 ± 0.21	*83.00* ± *0.15*	82.63
	EKRR	82.50 ± 0.09	*82.30* ± *0.12*	82.30 ± 0.14	82.37
	Ours	**84.19** ± **0.96**	**84.71** ± **0.38**	**85.05** ± **0.56**	**84.65**
E	DT	61.60 ± 0.25	63.00 ± 0.45	63.40 ± 0.56	62.67
	RF	76.90 ± 0.80	80.70 ± 0.10	81.10 ± 0.06	79.57
	NP	74.80 ± 0.30	71.90 ± 0.80	72.00 ± 0.80	72.90
	WP	*85.00* ± *0.30*	*85.30* ± *0.70*	*85.50* ± *0.12*	85.27
	NBI	84.90 ± 0.30	85.20 ± 0.70	85.40 ± 0.12	85.17
	RLS	76.70 ± 0.40	74.30 ± 0.10	74.40 ± 0.21	75.13
	RK	77.40 ± 0.13	75.90 ± 0.40	76.20 ± 0.17	76.50
	EDT	82.20 ± 0.20	84.50 ± 0.30	84.70 ± 0.80	83.80
	EKRR	77.30 ± 0.50	75.30 ± 0.24	75.80 ± 0.38	76.13
	Ours	**85.83** ± **0.47**	**87.95** ± **0.36**	**88.10** ± **0.53**	**87.29**
DB	DT	65.70 ± 0.48	69.30 ± 0.64	70.30 ± 0.58	68.43
	RF	82.50 ± 0.60	85.30 ± 0.56	86.50 ± 0.76	84.77
	NP	69.70 ± 0.78	72.50 ± 0.46	72.90 ± 0.80	71.70
	WP	84.20 ± 0.64	78.20 ± 0.62	73.30 ± 0.48	78.57
	NBI	64.40 ± 0.93	58.90 ± 0.79	55.90 ± 0.53	59.73
	RLS	88.10 ± 0.75	87.70 ± 0.39	87.30 ± 0.35	87.70
	RK	88.30 ± 0.66	89.00 ± 0.60	88.70 ± 0.65	88.67
	EDT	87.50 ± 0.58	88.32 ± 0.62	88.89 ± 0.49	88.24
	EKRR	*91.00* ± *0.68*	*92.10* ± *0.86*	*92.40* ± *0.57*	91.83
	Ours	**92.38** ± **0.58**	**92.97** ± **0.72**	**93.25** ± **0.66**	**92.87**

The maximum and second maximum AUC are shown in bold and italics

As to the first problem, we compare AUCs of the compared methods when the threshold $\tau$ is set to 1, 3 and 5, which is given in 3–5 columns in Table 3. Setting $\tau$ to different values can show the adaptability of our algorithm. It can be seen from Table 3 that the AUCs of our method are all the best. Specifically, AUCs of our method are respectively 2.47%, 0%, 1.49%, 0.83% and 1.38% higher than that of the second best method when $\tau = 1$, where the second best method are EDT, WP, EDT, WP and EKRR on NR, IC, GPCR, E and DB. AUCs of our method are respectively 2.32%, 1.48%, 2.41%, 2.65% and 0.87% higher than that of the second best method when $\tau = 3$, where the second best method are RK, WP, EKRR, WP and EKRR on NR, IC, GPCR, E and DB. AUCs of our method are respectively 1.84%, 2.31%, 2.05%, 2.60% and 0.85% higher than that of the second best method when $\tau = 5$, where the second best method are EKRR, WP, EDT, WP and EKRR on NR, IC, GPCR, E and DB. It can be seen from the above results that our method is obviously better than the compared methods regardless which value is set to $\tau$ and much better than the compared methods when $\tau$ is set to 3 and 5.

**Fig. 4:**
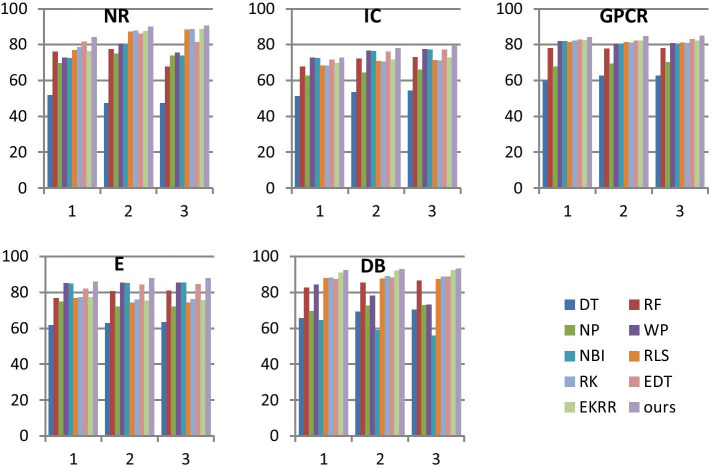
Histogram of AUCs for TWLNI, where $\tau$ used in Algorithm.1 are respectively set to 1, 3 and 5

Furthermore, to better show the results of methods with different $\tau$, the histogram form of Table 3 is given in Fig. 4. It can be seen form Fig. 4 that our method is obviously increased with the increase of $\tau$ on NR, IC, E and DB, but most of the compared algorithms have no similar phenomena. The reason may be that more positive samples will be generated with the increase of $\tau$ for a target, and then there would be enough positive samples to predict the interactions of this target. As a result, adding samples of neighbors may be worsening for predicting the DTI of this target.

As to the second problem, we will answer it from three aspects. Firstly, it can be seen from Table 3 and Fig. 4 that our method is the best regardless which value is set to $\tau$ on all datasets. Specifically, it can be seen from the last column in Table 3 that our method is the best method on all 5 datasets, and the AUCs of our method are respectively 3.31%, 1.27%, 2.02%, 2.02% and 1.04% higher than the second best methods on NR, IC, GPCR, E and DB, where the second best methods are respectively RK, WP, EDT, WP and EKRR. They prove that our method owns the best effect for DTI predicting. Secondly, it can be seen from Table 3 and Fig. 4 that the second best methods are very different on different datasets or by setting different value for $\tau$. It proves that our method is more stable than the compared methods. Thirdly, it can be seen from Fig. 4 that our method is obviously increased with the increase of $\tau$ on most datasets, which provides a very good guide to the scope of application of our algorithm. As a result, our method is much better than the compared methods.

### The experiments for TWSNI

The experiment results are presented in Table 4. These experiments would be used to answer the following questions:

1. Which threshold $\tau$ should be set for our method?
2. Is our method better than the compared methods?

**Table 4:** AUCs for TWSNI, where $\tau$ used in Algorithm 1 are respectively set to 1, 3 and 5

Dataset	$\tau$	1	3	5	Mean
NR	DT	49.90 ± 0.54	54.30 ± 0.32	53.20 ± 0.31	52.47
	RF	51.10 ± 0.61	63.90 ± 0.51	66.40 ± 0.44	60.47
	WP	32.30 ± 0.64	53.90 ± 0.58	58.30 ± 0.44	48.17
	NBI	40.20 ± 0.54	54.80 ± 0.60	58.60 ± 0.44	51.20
	RLS	52.90 ± 0.65	**66.20** ± **0.49**	**69.90** ± **0.48**	**63.00**
	RK	49.10 ± 0.63	66.00 ± 0.51	69.40 ± 0.48	61.50
	EDT	44.70 ± 0.73	63.00 ± 0.70	66.70 ± 0.51	58.13
	EKRR	51.00 ± 0.59	66.20 ± 0.52	69.60 ± 0.45	62.67
	**ours**	**53.90** ± **0.47**	65.30 ± 0.52	67.90 ± 0.33	62.37
IC	DT	46.70 ± 0.70	48.70 ± 0.41	50.30 ± 0.43	48.57
	RF	41.90 ± 0.48	54.60 ± 0.34	61.50 ± 0.33	52.67
	WP	37.40 ± 0.59	50.80 ± 0.55	60.70 ± 0.45	49.63
	NBI	32.40 ± 0.69	50.40 ± 0.57	60.20 ± 0.44	47.67
	RLS	51.30 ± 0.73	58.80 ± 0.41	63.00 ± 0.32	57.70
	RK	46.40 ± 0.91	**60.50** ± **0.40**	65.30 ± 0.32	57.40
	EDT	34.10 ± 0.58	54.60 ± 0.49	62.90 ± 0.39	50.53
	EKRR	50.90 ± 0.76	60.10 ± 0.53	**65.50** ± **0.42**	**58.83**
	ours	**54.50** ± **0.51**	54.40 ± 0.34	57.20 ± 0.36	55.37
GPCR	DT	50.50 ± 0.58	53.10 ± 0.37	57.30 ± 0.28	53.63
	RF	56.80 ± 0.68	69.40 ± 0.32	73.50 ± 0.26	66.57
	WP	40.80 ± 0.68	58.40 ± 0.30	64.10 ± 0.21	54.43
	NBI	40.80 ± 0.65	58.00 ± 0.29	64.10 ± 0.20	54.30
	RLS	71.90 ± 0.53	80.40 ± 0.30	81.50 ± 0.23	77.93
	RK	71.80 ± 0.56	79.20 ± 0.32	80.40 ± 0.25	77.13
	EDT	55.80 ± 0.52	70.00 ± 0.30	74.80 ± 0.23	66.87
	EKRR	**72.50** ± **0.48**	**80.80** ± **0.26**	**82.00** ± **0.20**	78.43
	ours	51.70 ± 0.28	59.60 ± 0.21	63.00 ± 0.13	58.10
E	DT	58.90 ± 0.18	61.40 ± 0.14	61.00 ± 0.21	60.43
	RF	58.90 ± 0.27	64.10 ± 0.17	67.90 ± 0.18	63.63
	WP	44.20 ± 0.21	47.40 ± 0.22	56.90 ± 0.29	49.50
	NBI	44.70 ± 0.28	47.60 ± 0.16	57.10 ± 0.32	49.80
	RLS	67.50 ± 0.22	68.30 ± 0.22	72.60 ± 0.24	69.47
	RK	67.00 ± 0.27	70.30 ± 0.27	**72.90** ± **0.29**	70.07
	EDT	58.00 ± 0.28	63.10 ± 0.19	68.30 ± 0.23	63.13
	EKRR	64.20 ± 0.22	65.70 ± 0.22	71.20 ± 0.25	67.03
	ours	**70.20** ± **0.19**	**71.70** ± **0.12**	69.70 ± 0.40	70.53
DB	DT	51.20 ± 0.52	57.30 ± 0.37	58.80 ± 0.39	55.77
	RF	54.40 ± 0.65	58.60 ± 0.68	62.20 ± 0.75	58.40
	WP	56.70 ± 0.37	45.10 ± 0.46	53.00 ± 0.38	51.60
	NBI	60.80 ± 0.45	60.20 ± 0.57	59.80 ± 0.57	60.27
	RLS	43.50 ± 0.76	51.60 ± 0.62	65.80 ± 0.63	53.63
	RK	**65.40** ± **0.59**	**70.50** ± **0.65**	**73.70** ± **0.68**	69.87
	EDT	52.39 ± 0.62	55.60 ± 0.49	63.30 ± 0.54	57.10
	EKRR	39.90 ± 0.38	53.30 ± 0.38	60.5. ± 0.38	51.23
	ours	62.50 ± 0.62	63.70 ± 0.67	64.50 ± 0.57	63.57

The maximum and second maximum AUC are shown in bold and italics

As to the first problem, we compare the AUCs of the compared methods when the threshold $\tau$ is set to 1, 3 and 5, which is given in 3–5 columns in Table 4. It can be seen from Table 4 that the AUCs of our method are the best on NR, IC, E, and the second best on DB when $\tau$ is set to 1. However, the AUCs of our method are worse than that of the most compared methods when $\tau$ is set to 3 and 5. Specifically, our method is much worse than compared methods when $\tau$ is set to 5.

**Fig. 5:**
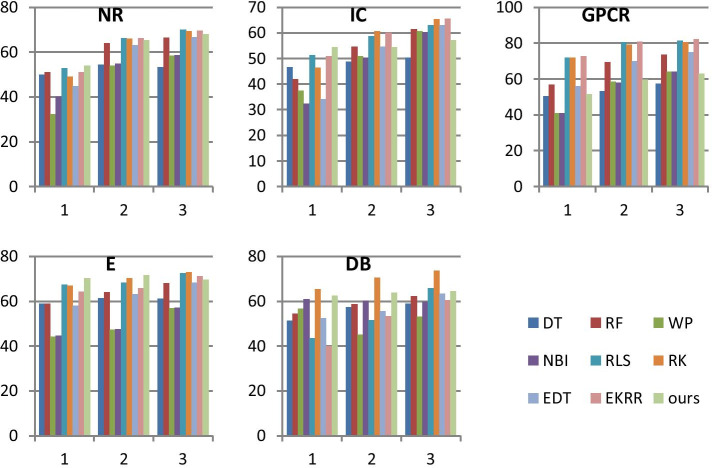
Histogram of AUCs for TWSNI, where $\tau$ used in Algorithm.1 are respectively set to 1, 3 and 5

Furthermore, to better show the results of algorithms with different $\tau$, the histogram form of Table 4 is given in Fig. 5. It can be seen from Fig. 5 that AUCs of almost all methods are obviously increased with the increase of $\tau$ on almost all datasets. However, the increase speed of our method is less than that of other methods. The reason may be that nearest profile is used to improve the DTI effect for TWSNI in this paper and nearest profile could be not very good for targets with a larger number of interactions.

As a result, $\tau$ should be set to 1 for our method. Although $\tau$ only can be set to 1, TWSNI classifier and TWSNI evaluator are also very useful and important. Firstly, the interaction of targets with a larger number of interactions can be predicted by TWLNI classifier and TWLNI evaluator. It can be seen from Table 3 that TWLNI classifier and TWLNI evaluator can obtain good results when $\tau$ is set to 3 and 5. Secondly, TWSNI classifier and TWSNI evaluator own good results when $\tau$ is set to 1. It can be seen from Table 4 that our method is the best on NR, IC, E, and the second best on DB when $\tau$ is set to 1. Thirdly, the best compared method for TWLNI and TWSNI are not the same, which prove that using different classifier strategies for different targets may be necessary. And then separately designed the TWSNI classifier and TWSNI evaluator for TWSNI is important. It can be seen from Tables 3 and 4 that the best method for TWSNI is RLS, but RLS is not the best method for TWLNI.

As to the second problem, we compare the AUCs of the compared methods when $\tau$ is set to 1, as TWSNI classifier and TWSNI evaluator are only used to improve the DTI effect for the targets with smaller numbers of interactions. It can be seen from the second column in Table 4 that our method is the best method on NR, IC and E, and the AUCs of our method are respectively 1.00%, 3.20% and 2.70% higher than that of the second best methods on NR, IC, and E, where the second best methods are RLS. It shows that our method is better than the compared methods on most datasets.

Furthermore, it can be seen from Table 4 that our method is worse than the most of the compared methods on GPCR, the reason may be that NP is used in our TWSNI classifier. NP can consider the problem that the similarity between drugs and the similarity between targets are not very precise, as only the nearest neighborhood is used to predict the DTI. However, this character also makes NP a little sensitive to the nearest neighborhood. As a result, our method can own good results on most datasets but owns bad result on GPCR. So if using our method to predict the DTI for TWSNI, many cross validation on training data should be firstly performance. Actually, most comparison algorithms are prone to the above phenomenon for TWSNI, as the positive samples are not enough for TWSNI. For example, RF is good on NR but bad on other three datasets. EKRR is the best on GPCR but not very good on other three datasets. RK is good on GPCR but not very good on NR and IC. As a result, our method can be also a good method to predict the DTI for targets with a small number of interactions in real applications.

## Discussion

Different targets are with very different numbers of interactions and most of the interactions focus on only a few targets. And then some targets could own enough positive samples to predict their interactions but other targets cannot just use their own positive samples to predict their interactions. As a result, for targets that own enough positive samples, the effect of predicting DTI could be worse by adding samples of neighbors, as neighbors could own much more negative samples than positive samples. However, for targets that do not have enough positive samples, many other positive samples should be utilized to improve the effect of DTI prediction. Obviously, the interactions of different targets should be predicted by different methods.

Furthermore, another problem is also existed in that different targets are with very different numbers of interactions. If TWSNI and TWLNI are evaluated together, the result could be mainly determined by TWLNI, as most of the interactions focus on only a few targets. However, finding new interactions for TWSNI could be more important than finding new interactions for TWLNI in the real application of the DTI prediction. Obviously, new evaluators should be designed to increase the influence of TWSNI on the results of the experiment.

In this study, MCSDTI is designed according above analyses, which owns following advantages: firstly, interactions of TWLNI and TWSNI are predicted by different classification strategies, which can make better use of the advantages of these classification strategies in different situations, and the information contained in different targets can be more fully utilized. Secondly, TWLNI and TWSNI are evaluated independently, and then the DTI prediction effect of TWSNI can be fairly presented, which provides a new research goal for DTI prediction. It can be seen from Tables 3 and 4 that MCSDTI is much better than the compared methods on most datasets. Specifically, most comparison methods cannot obtain good results for TWLNI and TWSNI in the same and many methods can own a good result for TWSNI but not for TWLNI. They prove that interactions for different targets should be predicted by different methods and all targets cannot be evaluated together.

There are several interesting problems to be investigated in our future work. Firstly, in this paper, an existed method is used to improve the DTI effect for TWSNI. Although this method can play its advantages under our framework, the DTI prediction result is also not very good, and then a better method can be designed for TWSNI in the future. Secondly, a new adaptively MCSDTI framework can be designed, where the number of parts can be adaptively chosen and the threshold used to divide the part can be adaptively set.

## Conclusions

This paper presents multiple classification strategies based drug-target interaction (MCSDTI) prediction method. In MCSDTI, targets are firstly divided into TWLNI and TWSNI; and then two classifiers and evaluators are respectively designed for TWLNI and TWSNI to predict the corresponding DTI. As a result, information of different target sets can be better used by different classification strategies; and the evaluation results obtained by different evaluation methods can fairer and more useful. The conducted experiments validate that MCSDTI is a competitive method compared to the previous ones. Most of methods cannot own both good DTI prediction results for TWLNI and TWSNI, but MCSDTI can be much better than the compared methods for both TWLNI and TWSNI on most datasets, which shows that designing different classification strategies for different targets is an effective way to improve the effectiveness of DTI prediction.

## Data Availability

The datasets processed for this article are freely available as described by [31] at http://web.kuicr.kyoto-u.ac.jp/supp/yoshi/drugtarget/ and freely available as described by [32] at https://go.drugbank.com/.

## Abbreviations

DTI: Drug-target interaction
MCSDTI: Multiple classification strategies based drug–target interaction
TWSNI: Targets with smaller numbers of interactions
TWLNI: Targets with larger numbers of interactions
NR: Nuclear receptors
IC: Ion channels
GPCR: G protein coupled receptors
E: Enzymes
DB: Drug bank
AUC: Area under the receiver operating characteristic curve
PSSM: Position-specific scoring matrix
XGBoost: Extreme gradient boosting
SMOTE: Synthetic minority oversampling technique
SIMILES: Simplified molecular input line entry system
PROFEAT: Protein features
NP: Nearest profile
DT: Decision tree
RF: Random forest
WP: Weighted profile
NBI: Network-based inference
RLS: Regularized least squares-avg
RK: Regularized least squares-kron
EDT: Ensemble decision tree
EKRR: Ensemble kernel ridge regression ensemble
MATLAB: Matrix laboratory
